# Secondary structure of protamine in sperm nuclei: an infrared spectroscopy study

**DOI:** 10.1186/1472-6807-11-14

**Published:** 2011-03-24

**Authors:** Alicia Roque, Inma Ponte, Pedro Suau

**Affiliations:** 1 Departamento de Bioquímica y Biología Molecular, Facultad de Biociencias, Universidad Autónoma de Barcelona, 08193 Bellaterra (Cerdanyola del Vallès), Barcelona, Spain

## Abstract

**Background:**

Protamines are small basic proteins that condense the DNA in mature spermatozoa. Typical protamines are of simple composition and very arginine-rich, usually in the range of 60-80%. Arginine residues are distributed in a number of stretches separated by neutral amino acids. We have used Fourier transform infrared spectroscopy (FTIR) to gain access for the first time to the secondary structure of protamines in sperm nuclei. This technique is particularly well suited to the study of DNA-bound protamine in whole nuclei since it is not affected by turbidity.

**Results:**

We show that DNA -bound salmon (salmine) and squid protamines contain *α*-helix, *β*-turns and a proportion of other structures not stabilized by intramolecular hydrogen bonding. No *β*-sheet was observed. In salmine, the *α*-helix amounted to ~20%, while in squid protamine it reached ~40%. In contrast, the structure not stabilized by intermolecular hydrogen bonding was more abundant in salmine (~40%) than in squid protamine (~20%). Both protamines contained ~40% *β*-turns. The different helical potential of salmine and squid protamine was confirmed by structure predictions and CD in the presence of trifluoroethanol.

**Conclusion:**

DNA-bound protamine in sperm nuclei contains large amounts of defined secondary structure stabilized by intramolecular hydrogen bonding. Both salmine and squid protamine contain similar amounts of *β*-turns, but differ in the proportions of *α*-helix and non-hydrogen bonded conformations. In spite of the large differences in the proportions of secondary structure motifs between salmon and squid protamines, they appear to be equally efficient in promoting tight hexagonal packing of the DNA molecules in sperm nuclei.

## Background

Protamines are small basic proteins which condense the DNA in mature spermatozoa [1]. The extremely compact nature of the nucleoprotamine complex and the inherent problems in the crystallographic approach have made it difficult to study the structure of DNA-bound protamine; as a result, several structural aspects of nucleoprotamine remain unsettled. Typical protamines are of simple composition and very arginine-rich, usually in the range 60-80%. They are characterized by a number of stretches of arginine residues separated by neutral amino acids. Fibre-diffraction diagrams from reconstituted nucleoprotamine and whole sperm cells indicate that DNA molecules are tightly packed in a hexagonal unit cell and that DNA is in a B-like structure, with ten base-pairs per helical turn [2-6]. The structural features of DNA-bound protamine are more difficult to establish because the protein, in contrast to DNA, is not sufficiently ordered to be visible in fibre diffraction diagrams.

There are two main types of model for the conformation of DNA-bound protamine: a) Those that assume that the protamine follows the path of either the narrow or the wide groove of the DNA, with the guanidinium groups of consecutive arginines binding alternatively to the phosphate groups of either strand of the DNA double helix [2,7]. In this models, the stretches of arginine lack intramolecular hydrogen bonding, and, therefore, of secondary structure of its own. This model is supported basically by model building studies. b) Other models assume that the stretches of arginine adopt an *α*-helical structure when bound to DNA. This model is supported mainly by the results of Warrant and Kim [8] showing that salmine (salmon protamine) adopts, at least in part, an *α*-helical conformation when diffused inside a preformed t-RNA crystal. A different kind of helical structure, the *γ*-ribbon, stabilized by 1→3 hydrogen bonding, has also been suggested for the DNA-bound protamine [9].

We have studied for the first time the secondary structure of salmine and squid protamine inside sperm nuclei by FTIR (Fourier transform infrared spectroscopy). This technique is particularly well suited to the study of DNA-bound protamine in whole nuclei since it is not affected by turbidity. In sperm nuclei, the DNA-bound protamine appeared to be structured by intramolecular hydrogen bonding to a large extent. The *α*-helix was present in both protamines, but it was more abundant in squid protamine than in salmine (~40% vs. ~20%). Other components were assigned to *β*-turns and to structures not stabilized by intramolecular hydrogen bonding.

## Results

### Structure predictions

**Figure 1:**
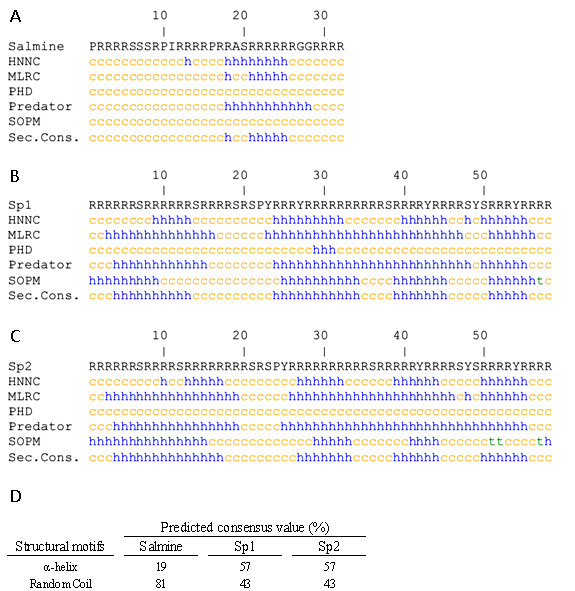
**Structure predictions for salmine and squid protamine using different methods**. Helical structure, turns and random coil are denoted by h, t and c, respectively. The last line shows the consensus prediction for salmine (A) and the Sp1 (B) and Sp2 (C) variants of squid protamine. (D) Proportions of secondary structure motifs for the consensus prediction for salmine, Sp1 and Sp2.

Salmine is a typical fish protamine of 32 amino acids, with 67% arginine. Squid protamine has two very similar components, Sp1 and Sp2 [10]. Sp2 is the most abundant (~75%), with 58 amino acids and 79% arginine. Squid protamines contain 4 (Sp2) or 5 (Sp1) tyrosines. The amino acid sequences are shown in Figure 1.

Secondary structure predictions for salmine and squid protamine were performed using several methods based on different principles (Figure 1). These methods were capable of predicting the presence of *α*-helix in both protamines. The consensus prediction for salmine contained 19% *α*-helix, while squid protamine (Sp1 and Sp2 components) showed a much higher helical propensity with 57% *α*-helix. Previously, the presence of α-helix in clupeine Z, a fish protamine, similar to salmine, was predicted with a modified version of the Chou and Fasman program [11,12].

### Circular dichroism

**Figure 2:**
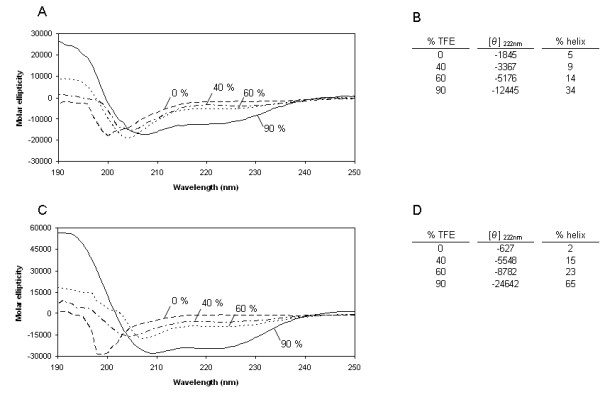
**CD spectra of salmine and squid protamine in trifluoroethanol**. (A) CD spectra of salmine. (C) CD spectra of squid protamine. The numbers refer to the TFE concentration in percentage by volume. The buffer was 10 mM HEPES, 140 mM NaCl, pH 7.0 at 20°C. Variation of the mean molar ellipticity ([*θ*], deg.cm^2^/dmol) at 222 nm with added TFE for salmine (B) and squid protamine (D). The percentage of helical structure, calculated as described in Methods is also indicated.

We studied the secondary structure of salmine and squid protamine by CD in the presence of trifluoroethanol (TFE), a widely used secondary structure stabilizer. Both protamines had little structure in dilute solution and physiological salt (140 mM NaCl). Addition of TFE increased the *α*-helical content, as shown by the increase of negative ellipticity at 222 nm and the change in the shape of the spectrum. The helical content of the protamines in function of TFE concentration was estimated by the method of Chen et al. [13] (Figure 2). In 90% TFE, salmine and squid protamine contained 34% and 65% *α*-helix, respectively. The absence of an isodichroic point in the spectra obtained in increasing TFE concentrations suggests the presence of regions of different helical propensity in both protamines. In 100% chloroethanol, a 51% of α-helix was reported for clupeine Z [14].

### Infrared spectroscopy of sperm nuclei

We studied the structure of DNA-bound salmine and squid protamine in purified sperm nuclei by infrared (IR) spectroscopy. The nuclei of mature sperm cells containing typical protamines, as salmine and squid protamine, do not appear to contain other protein components [15-20]. To confirm the absence of non-protamine proteins in nuclei in amounts that could compromise the attribution of the IR signal to protamine, total nuclear extracts were obtained and analyzed as described in Methods. Combined SDS and urea/acetic acid gel electrophoresis showed that protamine was practically the only protein component of nuclei (Additional file 1: Figure S1).

**Figure 3:**
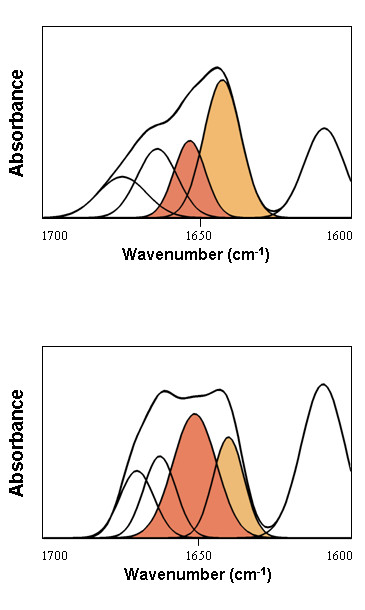
**Amide I' decomposition of salmine and squid protamine in purified sperm nuclei**. The DNA contribution was subtracted as described in Methods. Spectra were measured in D_2_O. The buffer was 10 mM HEPES plus 140 mM NaCl, pH 7.0 at 20°C. The concentration of nuclei expressed as DNA concentration was 5 mg/ml. The peak at 1609 cm^-1 ^corresponds to the arginine side chain plus, in the case of squid protamine (lower panel), a contribution from tyrosine side chains. The *α*-helix component is highlighted in red and the random coil/flexible regions are highlighted in orange.

Nuclei were analyzed immediately after purification and sample drying was avoided. The number and position of the amide I' (D_2_O) band components were obtained by Fourier deconvolution and used for the curve fitting of the original envelope by an iterative process previously described [21]. Measurements were performed at concentration expressed as DNA concentration of 5.0 mg/ml (Figure 3) and 25 mg/ml with virtually identical results indicating lack of concentration dependence of the spectra (data not shown).

In sperm nuclei and physiological salt (140 mM NaCl), salmine had a component band representing 20% of the total amide I' intensity at 1652 cm^-1^, which is the canonical position of the *α*-helix. Two other component bands at 1674 cm^-1 ^(16%) and 1663 cm^-1 ^(22%) were assigned to *β*-turns. The main amide I' component of salmine was at 1642 cm^-1^, with 42% of the total intensity (Figure 3 Table 1). Vibrations in this region are usually assigned to random coil/flexible regions, lacking stable patterns of intramolecular hydrogen bonding. A component at about 1640 cm^-1 ^was already observed in early studies of nucleoprotamine films [22]. The extended conformations imposed by the DNA template, assumed by some models, lack intramolecular hydrogen bonding and would be compatible with the component band at 1640 cm^-1^, but considering that the percentage of this component is lower than the percentage of arginine, at least part of the arginine residues should be present as *α*-helix or *β*-turns.

**Table 1:** Percentages of secondary structure of salmon and squid protamines in sperm nuclei.

	Salmon protamine		Squid protamine			
		
Assignment	140 mM		140 mM		600 mM	
			
	Band cm^-1^	%	Band cm^-1^	%	Band cm^-1^	%
Turns	1674	16	1669	17	1669	17
Turns	1663	22	1662	20	1661	22
α-helix	1652	20	1650	40	1650	39
Random coil/flexible regions	1642	42	1640	23	1640	22
Side Chain	1609	-	1609	-	1609	-

The concentration of nuclei expressed as DNA concentration was 5 mg/ml. Band position (cm^-1^) and percentage area (%) and assignment of the components were obtained after curve fitting of the amide I band (D_2_O). The buffer was 10 mM Hepes plus 140 or 600 mM NaCl, pH7.0. The values were rounded off to the nearest integer.

Deconvolution of the amide I' of squid protamine in sperm nuclei gave the same components observed in salmine. The *β*-turn components had similar proportions to those in salmine: 17% at 1669 cm^-1 ^and 20% at 1662 cm^-1^. In contrast, the *α*-helix was dominant, with 40% of the total intensity, while the component of random coil/flexible regions decreased to 23%. *β*-sheet components were not observed neither in salmine nor in squid protamine (Figure 3 Table 1).

Squid sperm nuclei were examined in both 140 mM and 600 mM NaCl, the latter corresponding to the salt concentration of sea water, with virtually identical results (Table 1).

The spectra of both protamines in sperm nuclei showed a large band at 1609 cm^-1 ^corresponding to the absorption of the side chains of arginine residues in D_2_O. The band was larger in squid protamine than in salmine, due to the higher content of arginine in squid protamine, but also to the contribution of the absorption at 1614 cm^-1 ^of the tyrosine side chains present in squid protamine [23] (Figure 3).

## Discussion

Protamines have little structure in dilute solution and physiological salt, but acquire different proportions of helical structure in the presence of TFE, a solvent that reveals the conformational propensities of polypeptides (Figure 2). Structure predictions using different programs based on different principles also indicate that protamines may have helical potential (Figure 1). These observations, although suggesting that protamines may have its own secondary structure when bound to DNA, do not give information on the representation of the different secondary structure motifs present in sperm nuclei. With the purpose of clarifying the issue, we examined purified sperm nuclei by IR spectroscopy.

The IR spectra of both DNA-bound protamines could be deconvoluted in terms of three secondary structure motifs: turns, α-helix and unordered structure. No intra or intermolecular *β*-sheet components were observed. Salmine contained 20% *α*-helix at the canonical position of 1652 cm^-1 ^and 38% *β*-turns (at 1674 cm^-1 ^and 1663 cm^-1^). The remaining 42% vibrated at 1642 cm^-1 ^(Table 1). This frequency is usually assigned (in D_2_O) to the unperturbed amide group interacting with solvent; i.e., not involved in intrachain or interchain hydrogen bonding, and is referred to as random coil/flexible regions. In salmine, the joint contributions of the secondary structure motifs stabilized by intramolecular hydrogen bonding (helix plus turns) thus amounted to ~60% of the sequence, while the remaining ~40% appeared as unstructured. Squid protamine was more extensively structured, the component band of random coil/flexible regions at 1640 cm^-1 ^representing only 23% of the total intensity. The *α*-helix was the major component with 40% of the total intensity. *β*-turns were the other main component with 37%, a percentage similar to that observed in salmine (Table 1).

Examination of the amino acid sequences shows that arginine tracks are shorter in salmine than in squid protamine (Figure 1). This fact, together with the higher proline content of salmine (three prolines out of 32 amino acids) compared to squid protamine (one proline out of 58 amino acids) may explain the higher content of *α*-helix in squid protamine than in salmine. With ~20% *α*-helix and 32 residues, salmine may have a single helical element, as indicated by structure predictions, while squid protamine with ~40% *α*-helix and 58 residues could contain two or more helical elements.

Models of nucleoprotamine have in common the hexagonal packing of the DNA molecules, but differ in the conformation and location of the protamine in the complex. In one model, the protamine winds around the minor groove of the DNA double helix with the side-chains of the arginine residues neutralizing the phosphate groups of DNA [2]. Similar models locate the arginine tracks in the DNA major groove on account of steric considerations [7], with the neutral amino acids protruding into the minor groove of neighbouring DNA molecules [7,24]. A main feature of such models is the absence of intramolecular hydrogen bonding involving the amide groups of the protein backbone.

Other models consider the structure of the DNA-bound protamine as composed of helical segments corresponding to the tracks of consecutive arginines connected by neutral amino acids. In some models the helical segments wrap around the DNA major groove [8], while in others they fill the space between the hexagonally packed DNA molecules [25-27]. These later models elaborate the early proposal of Luzzati and Nicolaiev [28], where protamine, in a non-specified conformation, fills the gaps between hexagonally packed DNA molecules.

In general, models for nucleoprotamine assume a uniform secondary structure for the arginine tracks and even for the entire protein. Our results show that the secondary structure of protamines in sperm nuclei is heterogeneous, and contains *α*-helix, *β*-turns and non-hydrogen bonded conformations. Furthermore, comparison of the experimental percentages of secondary structure with the percentages of arginine in salmine and squid protamine indicates that all the arginine residues cannot be in a single conformation, since the percentages of *α*-helix are lower than those of arginine in both protamines, and the same applies to random coil/flexible regions and *β*-turns. The results showing large differences in secondary structure between salmine and squid protamine indicate that there is not a unique conformation for protamine in sperm nuclei, in spite of the common hexagonal packing of DNA molecules.

## Conclusion

The possibility of deconvoluting the amide I' band in components arising from different secondary structure motifs, together with the insensibility of IR spectroscopy to light scattering artefacts, has allowed to show that in sperm nuclei protamines contain large amounts of defined secondary structure stabilized by intramolecular hydrogen bonding. Both salmine and squid protamine contain similar amounts of *β*-turns, but differ in the proportions of *α*-helix and non-hydrogen bonded structure. It is to be noted that in spite of these conformational differences, both protamines are equally efficient in promoting tight hexagonal packing of the DNA molecules [2,5].

## Methods

### Purification of spermatozoa cell nuclei

Spermatozoa from salmon (*Onchorhyncus keta*) were centrifuged at 1800 g for 5 minutes and resuspended in 10 mM HEPES plus 140 mM NaCl, pH 7 (buffer A). Resuspended spermatozoa were sonicated three times for 30 seconds on ice, with 30 seconds intervals between each burst, using a Branson Sonifier 450 fitted with a microtip probe, at a power setting of 5, and then centrifuged at 1800 g for 5 minutes. The pellet was resuspended in buffer A plus 0.5% (v/v) Triton X-100, incubated at room temperature for 15 minutes and then homogenized with a glass DOUNCE tissue homogenizer. The homogenized preparation containing the nuclei was washed twice with buffer A and finally resuspended in 20 ml of buffer A containing 1 M sucrose. The suspension was placed above 10 ml of buffer A containing 2 M sucrose and ultracentrifuged at 68000 g for 90 minutes in a Beckman Coulter Optimum™L-100 XP ultracentrifuge.

Squid (*Loligo pealeii*) spermatophores were minced with scissors in 600 mM NaCl to liberate the spermatozoa. The spermatozoa were recovered by centrifugation at 1800 g for 5 minutes. Cell nuclei were purified with the same protocol used to purify salmon sperm nuclei.

Both salmon and squid nuclei were virtually 100% pure as judged by phase-contrast microscopy.

### Protamine purification

Salmon and squid sperm nuclei were extracted overnight with 5% acetic acid. The supernatant was discarded and the sediment was extracted with 0.25 M HCl for 14 h. Protamines were recovered from the extract by precipitation with six volumes of acetone and dried under vacuum. The purity of the proteins was assessed by urea/acetic acid gel electrophoresis (Additional File 1: Figure S1).

### Protein composition of sperm nuclei

Purified salmon sperm nuclei were dissociated with 2 M NaCl and squid sperm nuclei with 2 M guanidine hydrochloride. Chromosomal DNA was pelleted by ultracentrifugation at 335000 g for 3 h in a Beckman Coulter OptimumTM L-100 XP ultracentrifuge. The proteins in the supernantant were precipitated with cold acetone (6:1) (v/v) and centrifuged at 16000 g for 15 min. The pellet was washed with 70% ethanol and dried in a Speedvaccum (Savant). The composition of the extracted proteins was assessed by a combination of SDS [29] and urea/acetic acid gel electrophoresis. Squid protamine was not visible in SDS gels because it is insoluble in buffers containing 0.1% SDS [17] (Additional File 1: Figure S1).

### Urea/acetic acid polyacrylamide gel electrophoresis

Protamines were analyzed by urea/acetic acid polyacrylamide gel electrophoresis. Gels contained 18% acrylamide, 2.5 M urea and 0.7% acetic acid. The running buffer was 0.7% acetic acid. Samples were dissolved in 2.5 M urea, 0.7% acetic acid and 15% glycerol and run at 22 mA for 2.5 h at 4°C after a pre-electrophoresis for 1.5 h. Gels were stained overnight with 0.1% Coomassie blue R-250 in methanol: water: acetic acid (5:5:1) (v/v) and distained by diffusion in the same solvent.

### Secondary structure predictions

Secondary structure predictions were carried out using the Network Protein sequence analysis server available at http://npsa-pbil.ibcp.fr/[30]. We used five different prediction methods: HNN (Hierarchical Neural Network) [31], an optimized multivariate linear regression method embedded in a hierarchical and modular algorithm; MLRC (Multivariate Linear Regression Combination) [31], which combines the results of GORIV, SIMPA96 and SOPMA, post-processing the outputs of the individual methods and generating class posterior probability estimates; PHD [32], a neural network system that uses the evolutionary information held by a multiple sequence alignment; Predator [33], a method based on the nearest neighbor detection and SOPM (Self-Optimized Prediction Method) [34], based in the homologue method with optimized parameters. The input sequences were B02669 for salmon protamine and those previously reported by Wouters-Tyrou et al. [10] for squid protamine variants Sp1 and Sp2.

### Circular dichroism

Protamines were at 0.075 mg/ml in 10 mM HEPES buffer, pH 7.0, plus 140 mM NaCl. Samples in aqueous solution and 40%, 60% and 90% trifluoroethanol (TFE) (v/v) were prepared. Spectra were obtained on a Jacso J-715 spectrometer in 1 mm cells at 20°C. The results were analyzed with standard analysis software (JACSO) and expressed as mean residue molar ellipticity [*θ*]. The helical content was estimated from the ellipticity value at 222 nm (*θ*_222 _), according to the empirical equation of Chen et al. [13]: % helical content = 100[*θ*_222 _/-39,500 × (1-2.57/n)], where n is the number of peptide bonds.

### Infrared spectroscopy

Salmon and squid sperm nuclei samples were measured at an equivalent DNA concentration of 5 and 25 mg/ml in 10 mM HEPES, plus 140 mM NaCl, pH 7. Squid nuclei were also measured in 600 mM NaCl, the salt concentration of sea water. The samples were exchanged with D_2_O using successive steps of incubation with deuterated buffer and centrifugation at 3000 g for 3 min. An incubation step of three days in D_2_O was included. Measurements were performed on a 660 Varian spectrometer equipped with a MCT detector, using a demountable liquid cell with calcium fluoride windows and 50 *μ*m spacers. Typically, 1000 scans for each background and sample were collected and the spectra were obtained with a nominal resolution of 2 cm^-1^, at 22°C. Data treatment and band decomposition were as previously described [21]. The DNA contribution to the spectra of sperm nuclei was subtracted using a DNA sample of the same concentration; the DNA spectrum was weighted so as to cancel the symmetric component of the phosphate vibration at 1087 cm^-1 ^in the difference spectra as described in Vila et al. [35].

## Authors' Contributions

AR recorded and analyzed the CD and IR spectra and participated in the preparation and electrophoretic analysis of sperm nuclei. IP carried out the structural predictions and participated in the preparation and analysis of the composition sperm nuclei. AR, IP and PS were involved in the drafting of the manuscript. The project was conceived and jointly supervised by AR, IP and PS. All authors read and approved the final manuscript.

## Supplementary Material

Additional file 1 Figure S1: Electrophoretic analysis of salmon and squid proteins from purified sperm nuclei. Click here for file
