# Supplementary material for: Secondary structure of protamine in sperm nuclei: an infrared spectroscopy study
Source: BMC Struct Biol. 2011 Mar 24;11:14. doi: 10.1186/1472-6807-11-14 (PMC3073880; doi:10.1186/1472-6807-11-14)
Supplement: Additional file 1 — Figure S1: Electrophoretic analysis of salmon and squid proteins from purified sperm nuclei. [file 1472-6807-11-14-S1.PDF]

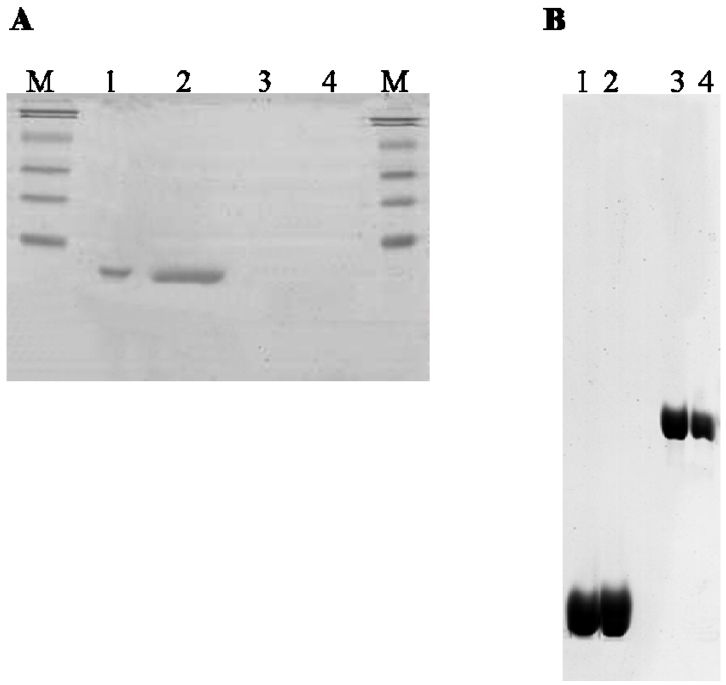

Figure S1. Electrophoretic analysis of salmon and squid proteins from purified sperm nuclei. A, SDS gel electrophoresis. B, urea/acetic acid gel electrophoresis. Purified protamine from salmon (Lane 1A and 1B) and squid (Lane 3A, 3B) sperm nuclei. Nuclear total proteins from salmon (Lane 2A and 2B) and squid (Lane 4A and 4B). Squid protamine was not observed in lanes 3A and 4A because it is not soluble in the presence of 0.1% SDS but it can be observed in the urea/acetic gel (3B and 4B). No proteins other than protamines were observed in total protein or acid extracts. M, Pre-stained molecular weight marker low range (BioRad).
